# In Vitro and In Situ Evaluation of Broccoli Wastes as Potential Feed for Ruminants

**DOI:** 10.3390/ani10111989

**Published:** 2020-10-29

**Authors:** Trinidad de Evan, Carlos N. Marcos, María José Ranilla, María Dolores Carro

**Affiliations:** 1 Departamento de Producción Agraria, Escuela Técnica Superior de Ingeniería Agronómica, Agroalimentaria y de Biosistemas, Universidad Politécnica de Madrid, Ciudad Universitaria, 28040 Madrid, Spain; t.deevan@alumnos.upm.es (T.d.E.); navarro-88@hotmail.com (C.N.M.); 2 Departamento de Producción Animal, Universidad de León, 24071 León, Spain. IGM (CSIC-ULE), Finca Marzanas s/n. 24346 Grulleros, León, Spain; mjrang@unileon.es

**Keywords:** broccoli, florets and stems, ruminal degradability, methane, intestinal digestibility, in vitro, in situ

## Abstract

**Simple Summary:**

Public concern about food wastes has increased in recent years. According to the FAO (Food and Agriculture Organization), vegetable food losses happen mainly at cultivation and harvest, but losses at distribution and consumption are also important. The dry matter of some vegetable wastes is rich in protein and fiber and the wastes could be used in ruminant feeding, but information on their nutritive value is needed. Both broccoli florets and stems were studied, and the rumen degradability of diets including increasing amounts of dried broccoli was assessed. Both florets and stems had low dry matter content (<5%), but were rich in protein (>23%) and sugars (>19.9%). Both broccoli fractions were highly degradable in the rumen, with stems showing greater values than florets. In contrast, stems had lower in vitro intestinal digestibility than florets. According to in vitro results, dried broccoli could replace up to 24% of the cereals and high-protein ingredients in a high-cereal concentrate without affecting the rumen fermentation of the diet.

**Abstract:**

The potential of broccoli wastes (florets and stems) as ruminant feed was analyzed using in vitro and in situ techniques. Both stems and florets had high moisture content (90.6 and 86.1%, respectively), but the stems contained (% dry matter) lower levels (*p* < 0.05) of crude protein (CP; 23.2 vs. 30.8%) and ether extract (2.91 vs. 6.15%) and tended to have greater sugars content (*p* = 0.071; 33.4 vs. 19.6%) than florets. Stems had greater in vitro dry matter rumen degradability (45.3%; 24 h incubation) and lower in vitro CP intestinal digestibility (82.7%) compared with florets (42.2 and 90.1%, respectively). Rumen degradability of protein was high (<85%) for both fractions. In a second experiment, diets including different proportions of broccoli were formulated and fermented in vitro. The replacement of 24% of conventional feeds (wheat, soybean meal and wheat bran) in a concentrate by dried broccoli increased the amount of organic matter fermented in vitro and the NH_3_-N concentrations of a mixed diet including 40% of the concentrate. Including dried broccoli in the diet produced only small modifications in the volatile fatty acid profile and did not affect CH_4_ emission.

## 1. Introduction

The production of broccoli (BRO; *Brassica oleracea* var. *italica)* has increased by 32.1% in the last decade, and in 2018 reached 37.2 × 10^6^ tons (production values combined with cauliflowers), with China and India being the main producers [1]. These two countries accounted for 81.1% of total worldwide production, and were followed by the United States of America, Mexico and Spain [1]. The marked increase in BRO production is mainly due to its relevance as a health-promoting food. The healthy attributes are ascribed to its high content in bioactive phytochemicals (glucosinolates, isothiocyanates, phenolic compounds) and nutrients, such as vitamins and minerals, and BRO consumption has proved to be beneficial for the prevention of chronic pathologies [2].

During the BRO supply chain for human consumption, there are multiple losses of vegetable material, which are generated during the agricultural production (cultivation and harvesting), processing, distribution, and consumption [3]. Values reported recently [4] indicate that in highly industrialized countries most vegetable losses happen at postharvest grading, which causes huge amounts of waste due to the high quality standards set by the retailers that can account for losses of about 45 to 50% of the BRO harvested [5]. In addition, the growing availability of frozen, canned and ready-to-eat vegetables is increasing the amount of waste of either whole vegetables or their fractions (i.e., leaves, stems). The potential use of BRO wastes as a source of bioactive compounds has been widely investigated [2,6,7], but their potential as ruminant feed has received less attention.

Similar to other vegetable wastes [8], the use of BRO wastes in ruminant feeding may reduce farming costs and the environmental contamination caused by their accumulation, as BRO wastes have high water content and are rapidly perishable. Several studies have analyzed the use of BRO wastes as feed ingredients in the diets of dairy cows [9], sheep [10], goats [11], and fattening lambs [12], and all of them obtained positive results. Furthermore, others have assessed the ensilability of BRO wastes either alone [13] or mixed with other feeds [12,14], and the in vitro ruminal fermentation of different BRO by-products [9,15,16,17,18]. However, only a single sample of BRO wastes was assessed in most of these studies, and none of them reported specific information on nutritive value of BRO florets. This study was therefore aimed to address the potential of BRO wastes (stems and florets) as feed for ruminants by measuring their chemical composition and their ruminal fermentation and intestinal digestibility by using in vitro methods. In addition, the possibility of using increasing amounts of dried BRO in sustainable ruminant diets was investigated by determining the in vitro ruminal fermentation, and the ruminal degradability of the diets was determined using the nylon bag technique.

## 2. Materials and Methods

The Institutional Animal Care and Use Committee of the Comunidad Autónoma de Madrid approved all the experimental procedures used in this study (Approval number PROEX 035/17), and animal care and handling followed the Spanish regulations for experimental animal protection.

### 2.1. Animals and Feeding

Four adult rumen-fistulated Lacaune sheep (64.3 ± 2.11 kg of body weight) were individually housed in floor pens with free access to fresh water over the study. Sheep were fed a mixed diet (2:1 grass hay:concentrate) at 45 g dry matter (DM)/kg body weight^0.75^. The ration was equally distributed in the morning (9:00 am) and in the afternoon (6:00 pm). The diet contained 114, 365 and 160 g of crude protein (CP), neutral detergent fiber (NDF) and acid detergent fiber (ADF) per kg DM, respectively.

### 2.2. Broccoli Wastes Samples

Three samples of BRO were obtained (one per week) in three different weeks between October and December 2017 from local markets. Each week, eight BRO pieces (about 0.5 kg weight) were collected from different markets and pooled. The vegetables from the same week were separated into stems and florets, and each fraction was weighed and pooled, before being chopped and dried at 40 °C until constant weight. In addition, two samples of feeds widely used in ruminant feeding, barley grains and wheat DDGS (dried distillers grains with solubles) were studied for comparative purposes. Fermentation parameters of both feeds have already been reported by de Evan et al. [3,19]. All samples were ground to 2 mm, and a subsample of each was taken and ground through a 1 mm sieve. Samples of 2-mm size were used for in situ incubations, and those of 1 mm for the rest of the measurements.

### 2.3. Experimental Design and Samplings

Two in vitro experiments were conducted using the same methodology. In the first experiment, chemical analyses of BRO wastes were conducted and in vitro methods were used to determine their degradability and digestibility. The objective of the second experiment was to assess the in vitro ruminal fermentation and ruminal degradation, measured in situ, of diets with variable proportions of dried BRO.

#### 2.3.1. Experiment 1: In Vitro Incubations of Broccoli Wastes

Two similar in vitro studies were conducted as described by De Evan et al. [19] to analyze the gas production kinetics and fermentative parameters of the samples. For both trials, 200 mg of DM of each sample (BRO stems and florets, barley grains and wheat DDGS) were carefully weighed into 60-mL glass vials. In addition, vials without substrate (blanks; two per inoculum) were included to correct for the endogenous gas production. To obtain the rumen fluid, rumen contents were collected from each sheep before the morning feeding and filtered through four layers of cheesecloth. The rumen fluid of each sheep was mixed in 1:4 proportion with culture medium [20], which was pre-warmed at 39 °C. The culture medium was modified by excluding the trypticase and replacing the (NH_4_)HCO_3_ with NaHCO_3_ to obtain a N-free medium. This procedure was followed to obtain four different replicates (i.e., sheep inoculum) per incubated sample. Twenty ml of the mixture were dosed in each vial by a Watson-Marlow 520UIP31 peristaltic pump (Watson-Marlow Fluid Technology Group, Cornwall, UK) under CO_2_ flushing. Vials were sealed with rubber stoppers and incubated at 39 °C. The first incubation lasted for 144 h, and the amount of gas produced was determined at different time intervals (3, 6, 9, 12, 15, 22, 26, 31, 36, 48, 58, 72, 96, 120 and 144 h after incubation) by a pressure transducer (Delta Ohm DTP704-2BGI, Herter Instruments SL, Barcelona, Spain) and a calibrated plastic syringe. The potential degradability of DM (PDDM) was estimated using an Ankom Daisy^II^ incubator (Ankom Technology Corp., Fairport, NY, USA). Three-hundred mg of each feed were weighed into filter bags (Ankom Corp #57; 25 µm pore size; Ankom Technology Corp., Fairport, NY, USA) in triplicate. Bags were incubated at 39 °C in a 1:4 mixture of ruminal fluid (mixture of all sheep) and the culture medium, as described previously [20]. After 144 h, bags were washed with cold water, dried at 60 °C for 48 h, and weighed to calculate the PDDM. This value was used to estimate the DM effective degradability (DMED), as described later.

The second in vitro incubation was conducted as described before and stopped after 24 h. After 24 h, gas production was measured, the content of the vials was homogenized and the pH was measured with a pHmeter Crison GPL 21 (Crison Instruments, Barcelona, Spain). Finally, 3 mL of vials content were mixed with 3 mL of 0.5 M HCl and frozen (at −20 °C) until volatile fatty acid (VFA) and NH_3_-N were determined.

The method of Gargallo et al. [21], which involves the use of the Daisy^II^ incubator, was followed to determine the in vitro intestinal digestibility of nitrogen (N) and DM of the BRO wastes. The details of the procedure have been described by De Evan et al. [3]. Briefly, BRO wastes were incubated in the rumen of each sheep into 46-μm pore size nylon bags for 12 h. Residues of incubation were pooled by BRO fraction and sheep, and 0.3 g were weighed in duplicate into Ankom R510 bags (dimensions 5 × 5 cm; 50 μm pore size). Bags were successively incubated in a pepsin-buffer solution (1 h) and in a pancreatin-phosphate buffer (24 h), washed and dried (40 °C; 72 h). Finally, the contents of the bags were mixed by BRO fraction and sheep before N analysis.

#### 2.3.2. Experiment 2: Ruminal Fermentation and Degradability of Diets with Dried Broccoli

The objective of Experiment 2 was to assess the potential of dried BRO to replace conventional feeds in ruminant diets. Five BRO pieces were obtained at local supermarkets and were cut into pieces, mixed, and dried at 40 °C until constant weight. The sample was ground to 2 mm for in situ incubations, and a subsample was ground to 1 mm for chemical analysis and in vitro incubations. All diets contained 40% of alfalfa hay and 60% concentrate (fresh matter basis). Four experimental concentrates were formulated: a high-cereal concentrate (control) and three additional concentrates in which different amounts of wheat, soybean meal and wheat bran were replaced by 8 (BRO8), 16 (BRO16) or 24 (BRO24) g of dried BRO per 100 g of concentrate. The concentrates were formulated to have similar crude protein (CP) and neutral detergent fiber (NDF) content.

Both the gas production kinetics and fermentation parameters of the diets were assessed in 144 and 24 h in vitro incubations, respectively, as described in Experiment 1. The 24 h incubation was performed in 120-mL glass vials, using 400 mg of sample DM and 40 mL of the ruminal fluid and culture medium mixture to get enough gas for CH_4_ analyses. After 8 h of incubation, gas production was measured, and a 10 mL sample of gas was collected into vacuum tubes for CH_4_ analysis. Immediately, 1 mL of vial content was taken using an insulin syringe, and was mixed with 1 mL of 0.5 M HCl and stored frozen (−20 °C) for VFA and NH_3_-N analyses. After 24 h, gas production was measured and samples for CH_4_ analysis were taken before opening the vials, measuring the pH of the content, and taking samples for VFA and NH_3_-N analyses.

The in situ measurement of DM and CP degradability of the diets followed the methodology described by de Evan et al. [3]. Briefly, 46-μm pore size nylon bags containing each diet (3 g) were incubated in the rumen of each sheep before the morning feeding and withdrawn after 2, 4, 8, 16, 24, 48 and 72 h. Two bags were incubated for each diet an incubation time, and each incubation series was replicated on different dates. Washing and processing of the bags were as described for the 12-h in situ incubations conducted in Experiment 1. In addition, 2 bags for each diet (0 h of incubation) were only washed using the same procedure to assess the insoluble fraction (washing losses). Bag residues were mixed by sheep before N analysis.

### 2.4. Chemical Analyses

The chemical composition of BRO fractions, barley grains, wheat DDGS, and feeds used in Experiment 2 was analyzed in duplicate. The procedures of AOAC [22] were used for the analysis of DM (ID 934.01), ash (ID 942.05) and ether extract (EE; ID 920.39). The sequential procedure described by Van Soest et al. [23] was utilized to determine the NDF and ADF content, and lignin content was determined as described by Robertson and Van Soest [24]. Results were expressed exclusive of residual ash. The Dumas combustion method and a Leco FP258 analyzer (Leco Corporation, St. Joseph, MI, USA) were used to analyze the N content of the samples and the neutral detergent insoluble CP (NDICP). Total sugar content was analyzed following the anthrone colorimetric method [25], employing an Epoch spectrophotometer (BioTek Instruents Inc., Winooski, VT, USA). Concentrations of NH_3_-N were determined by the phenol-hypochlorite method as described by Weatherburn [26], and those of VFA and CH_4_ by gas chromatography as described by García-Martínez et al. [27] and Martínez et al. [28], respectively.

### 2.5. Calculations and Statistical Analyses

The gas production data were fitted to the following exponential model Gas = A (1 − e ^(– c (t – *lag*))^) using the Proc NLIN of the SAS [29]. In this model, A is the potential or asymptotic gas production, *c* is the fractional gas production rate, *lag* is the time until gas production begins, and t is the gas measurement time. The average gas production rate (AGPR) is the gas production rate in the period from 0 to the time reaching half of the potential gas production and was estimated as proposed by France et al. [30]: AGPR = A *c* / [2 (ln2 + *c lag*)]. The DMED was estimated as: DMED = [(PDDM × *c*) / (*c* + *K*p)] e ^(^^−^*^kp^* ^×^^lag)^ for a *k*p (rumen passage rate) of 0.042 per h, which represents 24 h of digesta retention time in the rumen and is found in ruminants at medium levels of intake [31]. In addition, the production of acetate, propionate and butyrate in each vial was used to calculate the amount of apparently fermented organic matter (AFOM), as described by Demeyer [32].

Data on in situ DM and CP degradation of the diets in Experiment 2 were fitted with time (t) to the equation proposed by Ørskov and McDonald [33]: y = *a* + *b* (1 − e ^– *c* t^), in which *a* represents the soluble fraction, *b* is the insoluble degradable fraction and *c* represents the fractional degradation rate of *b*. The potentially degradable fraction was estimated as (a + *b*). Effective degradability (ED) of DM and CP was calculated according to the equation: ED = (a + b × *c*) / (*c* + kp) and using a Kp value of 0.042.

All statistical analyses were performed with the SAS package [29]. Data on chemical composition of BRO fractions in Experiment 1 were analyzed as a one-way analysis of variance, with the BRO fraction being the main effect. Gas production values and fermentation parameters data from Experiment 1 were analyzed using the PROC MIXED of SAS as a mixed model, in which the effect of the BRO fraction was considered fixed and that of the inoculum was considered random. Data on intestinal digestibility were analyzed using the same model, with the effect of the BRO fraction being fixed and that of the sheep used for the in situ incubations being random. Data from Experiment 2 were also analyzed as a mixed model, in which the inclusion of BRO (0, 8, 16 and 24% of the concentrate) was considered as a fixed effect and that of the rumen inoculum was random. In addition, non-orthogonal polynomial contrasts were used to analyze the linear and quadratic effects of including increasing levels of BRO in the diet. Values of *p* < 0.05 were considered statistically significant and those < 0.10 were considered trends. In Experiment 2, means were compared by Tukey’s test.

## 3. Results and Discussion

### 3.1. Experiment 1. Characteristics of Broccoli Fractions

**Figure 1:**
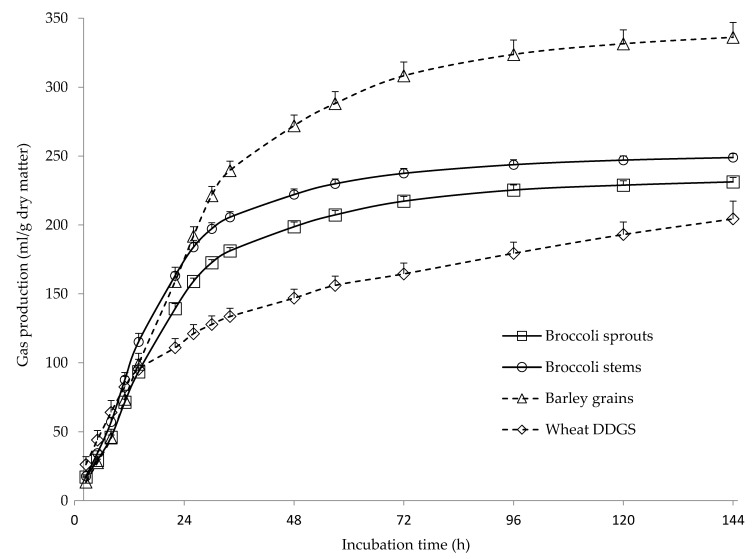
Gas production kinetics of broccoli sprouts, broccoli stems, barley grains and wheat DGGS (dried distilled grains with solubles). The bars indicate the standard error of the mean (*n* = 12 for broccoli samples, and *n* = 4 for barley grains and wheat DDGS).

The chemical composition of BRO stems and florets and of the reference feeds is shown in Table 1. The average proportions of stems and florets were 59.8 and 40.2%, respectively (as fed basis). As reported in previous studies on BRO and other vegetables of the *Brassicaceae* family [15,18,34], all samples had high moisture content, although the florets had greater DM content than the stems (*p* = 0.007; 13.9 and 9.41%, respectively). Compared to the stems, the florets contained greater (*p* ≤ 0.041) amounts of organic matter (OM), CP, EE, hemicellulose, non-soluble carbohydrates (NSC; calculated as 100 − [ash + CP + EE + NDF]) and NDICP, tended (*p* = 0.071) to have lower sugar amounts, and had lower (*p* = 0.044) ADF content. The OM content was similar to that reported in previous studies evaluating BRO stems [15,18]. However, BRO stems showed greater amounts of CP and EE, and lower amounts of NDF, ADF and lignin than those previously reported for dried stems [34], BRO by-products [35] and BRO stems preserved as hay or silage [14,18]. The composition of the stems in our study agrees well with the values reported by Sanarya et al. [36], who also observed differences in the chemical composition of BRO by-products (stems and leaves) produced in three different locations. As pointed out by Bakshi et al. [37], the chemical composition of BRO by-products might depend on their botanical origin, agroclimatic conditions, growth stage, the fraction of the plant included in the by-product and processing conditions. The content in OM, CP and EE of BRO florets is in accordance with previous values reported for this fraction [38,39]. Both BRO fractions and the reference feeds were incubated with sheep ruminal fluid to assess their gas production kinetics and fermentative parameters (Table 2 and Figure 1). Broccoli stems had greater (*p* < 0.001) A, *c* and AGPR values than the florets, but there were no differences (*p* = 0.763) between fractions in the time until the start of gas production (*lag*). These results indicate a faster and greater extent of degradation of the stems compared with the florets, as confirmed by the greater (*p* = 0.001) DMED values of the stems. The greater rumen degradation of the stems is consistent with their larger sugars content compared with the florets (33.4 and 19.6%, respectively), as sugars are rapidly and completely degraded by ruminal microorganisms [40], and the similar NDF content of the two fractions (22.3 and 23.6% for stems and florets, respectively). The high CP content of the florets (30.8 and 23.2% for florets and stems, respectively) might have contributed to their lower gas production, as protein fermentation generates less gas than that of carbohydrates [41]. De Evan et al. [19] used sheep ruminal fluid as inoculum in batch cultures to assess the gas production kinetic of cauliflower and romanesco fractions, and, in agreement with our results, observed greater A values for stems compared with florets, with no differences between fractions in *lag* values.

The A values agree well with those reported by Megías et al. [15] for BRO stems and by Marino et al. [16] for leftover BRO sampled at supermarkets. In contrast, Durmic et al. [17] reported greater A values (>400 mL/g DM) for BRO stems and leaves, and García-Rodríguez et al. [18] obtained lower A values (200 mL/g DM) for BRO stalk hay. Values of *c* in our study are in accordance with those previously reported for BRO wastes [15,18], although *lag* values were greater. As pointed out by Rymer et al. [42], there are many sources of variation (animal donors of rumen fluid and their feeding, culture medium, measurement equipment, etc.) that can influence the in vitro gas production, and direct comparison with results from other studies is difficult. Therefore, we included in the incubations a sample of barley grain and of wheat DDGS to be used as reference. The gas production of both BRO stems and florets was similar to that of the two reference feeds over the first 12 h of incubation, but at 24 h of incubation the BRO stems and barley grains showed greater gas production than BRO florets and wheat DDGS (Figure 1). From 30 h of incubation onwards, both BRO fractions produced lower gas than barley grains but more than wheat DDGS. These results are consistent with the A and AGPR values observed for both BRO fractions, being intermediate between those for barley grains and wheat DDGS.

Similar to that observed in the gas production kinetics incubations, broccoli stems produced more gas (*p* < 0.001) in the 24-h incubations than the florets (Table 2). Consistently, pH values were lower and total VFA production was greater (*p* < 0.001 for both) for the stems compared with the florets. Additionally, there were differences in the VFA profile, with stems producing more (*p* ≤ 0.014) acetate and butyrate and less (*p* < 0.001) minor VFA than the florets. During the deamination of branched amino acids, minor VFA are produced [43], and the lower proportion of minor VFA observed for the stems would indicate reduced CP degradation. This is consistent with both the lower CP content of the stems than the florets (23.2 vs. 30.8%, respectively) and the lower NH*_3_*-N concentrations (*p* < 0.001; 260 vs. 325 mg/L). Despite the significant differences between BRO fractions in VFA profile, their magnitude was small and both fractions had similar (*p* = 0.355) acetate:propionate ratios. Durmic et al. [17] analyzed the 24-h in vitro fermentation of a mixture of BRO leaves and stems and observed similar propionate proportions to those found in our study, but greater proportions of acetate and butyrate. Madrid et al. [34] reported much greater proportions of acetate (78.3%) and lower of propionate (13.7%) for 72-h in vitro incubations of BRO stems, but it should be taken into account that acetate proportions usually increase and those of propionate decrease with advancing incubation time [44]. Compared with the reference feeds, BRO stems showed greater total VFA production, but the VFA production of BRO florets was similar to the barley grains, which is in accordance with the similar DMED values observed for both of them (42.2 and 43.3%, respectively). The acetate:propionate ratio for barley grains was only slightly lower than that for BRO stems and florets. Both BRO fractions showed greater NH_3_-N concentrations than wheat DDGS, even though the CP content of wheat DDGS was similar to the BRO florets (Table 1). This could be explained by the lower proportion of NDICP in the BRO fractions than in the DDGS, which would indicate a high availability of BRO protein to rumen microorganisms.

The in situ rumen degradability and in vitro intestinal digestibility of BRO fractions are shown in Table 3. The high DM and CP rumen degradability (>85%) of both fractions after 12 h of in situ incubation confirm the in vitro results, indicating a rapid and extensive degradation of BRO by ruminal microorganisms. Rumen degradability of CP in BRO stems was 9.8% greater (*p* < 0.001) than in the florets, which is in accordance with their lower proportion of NDCIP (Table 1). Yi et al. [9] reported similar DM degradability values (88.2%) for pelletized BRO by-products after 24 h of in situ incubation in the rumen of sheep, but the CP degradability (50.2%) was lower than in our study (>85.3%). The in vitro intestinal digestibility of both DM and CP was lower (*p* ≤ 0.001) for BRO stems than for florets. Both fractions had low DM intestinal digestibility (<68%), which is in accordance with the high rumen degradability values, as the digesta flowing to the intestine is mainly composed of rumen-undegradable fractions. Our results agree well with the high CP intestinal digestibility values (88.9 to 93.2%) reported by de Evan et al. [19] for stems and florets from cauliflower and romanesco. In summary, both BRO fractions were rapidly degraded and contained low by-pass CP.

### 3.2. Experiment 2. Fermentation Parameters and Degradability of Diets with Dried Broccoli

The BRO samples (stems and florets) used in this experiment contained 89.3, 34.3, 27.0, 17.8 and 6.07 g of OM, CP, NDF, ADF and EE per 100 g of DM, respectively, and their chemical composition was in good agreement with results from Experiment 1. Ingredients and chemical composition of the diets are shown in Table 4. The dried BRO replaced different amounts of wheat grains, wheat bran and soybean meal, due to its high CP content and medium content of highly degradable NDF. As expected, all diets had the intended CP and NDF (16.1 and 31.5%, respectively).

As shown in Table 5, replacing conventional feeds by dried broccoli in the concentrate increased A and AGPR (*p* = 0.046 and 0.014; quadratic and linear, respectively), and tended to increase (*p* = 0.061; linear) the DMED of the diets, with no differences among diets in *c* and *lag* values. These results indicate a greater rumen degradability of BRO compared with the feeds that were replaced. The results of the 24-h incubations appear to support this hypothesis, as total VFA production and AFOM increased linearly (*p* < 0.001) at both 8 and 24 h of incubation with increasing BRO levels. The proportions of all individual VFA were also significantly changed at both sampling times. Acetate proportion showed a linear increase (*p* < 0.001), whereas that of propionate, butyrate and minor VFA showed a linear decrease (*p* ≤ 0.026; quadratic for minor VFA at 24 h), resulting in greater acetate:propionate ratios (*p* < 0.001) as the amount of BRO in the diet increased. Yi et al. [9] analyzed the in vitro fermentation of diets with pelleted BRO by-products (0, 10, 20, 30 and 40%) and observed similar changes in VFA profile and increases in the acetate:propionate ratio as the amount of BRO by-product in the diet augmented.

There were no differences (*p* ≥ 0.112) among diets at any sampling time in either CH_4_ production or CH_4_/VFA ratio, indicating a lack of antimethanogenic compounds in BRO. Similarly, Durmic et al. [17] reported no antimethanogenic activity of two samples of BRO with different organosulfur content in in vitro incubations. In accordance with the results of Yi et al. [9], the NH_3_-N concentration augmented linearly (*p* ≤ 0.002) at both incubation times as the amount of BRO in the diet increased, which agrees well with the high CP rumen degradability observed in Experiment 1. The in situ incubation of the diets (Table 6) confirmed these results, as increasing the level of BRO inclusion tended to linearly increase (*p* = 0.093) the ED of the dietary CP. Increasing the percentage of BRO inclusion in the diet linearly augmented (*p* ≤ 0.003) the soluble fraction (*a*) of both DM and CP, and linearly decreased (*p* ≤ 0.009) their non-soluble potential degradation fraction (*b*) without changes in the potential degradability of the diets (*a* + *b*). These results agree well with the high sugars content of BRO, and confirm the high rumen degradability of BRO protein.

## 4. Conclusions

Broccoli florets have greater dry matter content than the stems (13.9 vs. 9.6%), but both are rich in sugars and highly-degradable protein, and contain medium proportions of fiber with low lignin levels. Both broccoli fractions were rapidly fermented by rumen microorganisms, and their in vitro rumen degradability was similar to that of barley grains. The in vitro intestinal digestibility of dry matter was low (<68%), but that of protein was high (>82%). Under in vitro conditions, the substitution of 24% of conventional feeds by dried broccoli in the concentrate of isonitrogenous diets for dairy sheep increased the amount of both the organic matter fermented and the protein degraded, with only subtle changes in VFA profile. These results indicate that broccoli wastes have high nutritional value for ruminants, but their use in practical feeding would be limited by their high water content.

## Figures and Tables

**Table 1:** Chemical composition of broccoli fractions and of barley and wheat DDGS (dried distilled grains with solubles) samples ^1^.

Sample	Dry Matter (%)	g/100 g Dry Matter								Non Structural Carbohydrates ^2^	Lignin (% Neutral Detergent Fiber)	NDICP ^3^ (% Crude Protein)
		Organic Matter	Crude Protein	Ether Extract	Sugars	Neutral Detergent Fiber	Acid Detergent Fiber	Lignin	Hemicellulose			
Broccoli stems	9.41	89.3	23.2	2.91	33.4	22.3	15.0	1.46	7.27	31.3	6.49	5.77
Broccoli florets	13.9	91.8	30.8	6.15	19.6	23.6	12.9	0.84	10.7	40.9	3.53	9.94
SEM ^4^	0.27	1.52	0.18	3.991	0.27	0.55	0.52	0.283	0.066	2.32	1.118	0.929
*p* =	0.007	0.002	0.024	<0.001	0.071	0.159	0.044	0.193	<0.001	0.041	0.134	0.033
Reference feeds												
Barley grains	89.9	97.3	12.4	3.16	3.69	22.7	5.23	1.22	19.6	59.0	5.37	14.5
Wheat DDGS	92.2	95.5	32.9	4.61	6.97	29.5	11.2	3.33	18.3	28.5	11.2	28.9

^1^ Three different samples of each vegetable fraction were analyzed; ^2^ Calculated as 100 − [(100 − organic matter) + crude protein + ether extract + neutral detergent fiber]; ^3^ NDICP: crude protein insoluble in neutral detergent; ^4^ SEM: standard error of the mean.

**Table 2:** Parameters of gas production kinetics and the main fermentative parameters (24-h incubations) of broccoli fractions (*n* = 3) and of barley and wheat DDGS (dried distilled grains with solubles) samples (*n* = 1).

Sample	Gas Production Parameters ^1^					Fermentation Parameters ^2^								
	A (mL/g)	*c* (%/h)	*Lag* (h)	AGPR (mL/h)	DMED (%)	Gas (mL)	pH	Total VFA (µmol)	mol/100 mol				Ac/Pr (mol/mol)	NH_3_-N (mg/L)
									Acetate	Propionate	Butyrate	Minor VFA		
Broccoli stems	246	5.66	3.05	8.08	45.3	37.4	6.60	1736	63.1	23.4	8.90	4.58	2.70	260
Broccoli florets	228	4.84	2.98	6.60	42.2	34.3	6.70	1581	61.4	23.1	8.60	6.83	2.65	325
SEM ^3^	2.3	0.141	0.166	0.217	0.43	0.38	0.009	12.2	0.18	0.23	0.082	0.108	0.034	7.4
*p* =	<0.001	<0.001	0.763	<0.001	0.001	<0.001	<0.001	<0.001	<0.001	0.422	0.014	<0.001	0.355	<0.001
Reference feeds														
Barley grains	352	5.04	2.85	10.5	43.3	49.9	6.60	1452	56.6	22.2	17.5	3.70	2.55	156
Wheat DDGS	185	4.15	0.00	5.55	30.1	26.3	6.73	1311	53.4	33.3	6.34	6.96	1.61	223

^1^ See text for parameters description; DMED: dry matter effective degradability; ^2^ 200 mg of substrate dry matter; VFA: volatile fatty acids; Minor VFA included isobutyrate, isovalerate and valerate; ^3^ SEM: standard error of the mean.

**Table 3:** Values of degradability (12 h in situ incubation) and in vitro intestinal digestibility of broccoli fractions ^1^.

Sample	Dry Matter Rumen Degradability (%)	Crude Protein Rumen Degradability (%)	Dry Matter Intestinal Digestibility (%)	Crude Protein Intestinal Digestibility (%)
Broccoli stems	89.5	95.1	49.0	82.7
Broccoli florets	89.2	85.3	67.8	90.1
SEM ^2^	0.85	1.09	0.82	0.92
*p* =	0.829	<0.001	<0.001	0.001

^1^ Three different samples of each vegetable fraction were analyzed; ^2^ SEM: standard error of the mean.

**Table 4:** Ingredients and chemical composition of diets with variable proportions of dried broccoli (BRO; 8, 16 and 24% of concentrate) used in Experiment 2.

Item	Diet			
	Control	BRO8	BRO16	BRO24
Diet ingredients (g /100 g fresh matter)				
Alfalfa hay	40.0	40.0	40.0	40.0
Concentrate	60.0	60.0	60.0	60.0
Concentrate ingredients (g /100 g fresh matter)				
Broccoli	-	8.0	16.0	24.0
Corn	32.0	32.0	32.0	32.0
Barley	30.0	30.0	30.0	30.0
Wheat	15.0	12.0	10.0	7.5
Soybean meal 46%	14.0	10.5	7.5	4.5
Wheat bran	7.0	5.0	2.5	0.0
Calcium soap	1.0	1.0	1.0	1.0
Calcium carbonate	0.5	0.5	0.5	0.5
Mineral/vitamin premix	0.5	0.5	0.5	0.5
Chemical composition ^1^				
Dry matter	89.7	91.0	91.0	91.0
Organic matter	93.0	92.3	91.5	90.8
Crude protein	16.1	16.1	16.1	16.1
Neutral detergent fiber	31.5	31.6	31.6	31.7
Acid detergent fiber	15.9	16.3	16.6	17.0
Ether extract	4.18	4.29	4.37	4.47

^1^ Individual feeds were analyzed and diet composition was calculated and expressed as g/100 g dry matter (except dry matter; g/ 100 g diet).

**Table 5:** Gas production and fermentative parameters of experimental diets with dried broccoli (BRO) used in Experiment 2 ^1^.

Item	Diet				SEM ^3^	*p* =	
	Control	BRO8	BRO16	BRO24		Lineal	Quadratic
Gas production parameters ^2^							
A (mL/g DM)	280 ^a^	285 ^ab^	290 ^b^	282 ^a^	2.8	0.431	0.046
*c* (%/h)	3.90	3.90	4.00	4.00	0.000	0.119	0.823
*Lag* (h)	1.10	0.93	0.90	0.76	0.129	0.102	0.881
AGPR (mL/h)	7.40 ^a^	7.57 ^ab^	7.91 ^b^	7.77 ^b^	0.107	0.014	0.195
DMED (%)	39.7	39.3	39.6	40.6	0.32	0.061	0.075
Fermentative parameters (8-h incubation)							
Total volatile fatty acids (VFA; µmol per vial)	1284 ^a^	1343 ^a^	1413 ^b^	1478 ^b^	20.5	<0.001	0.887
Individual VFA (mol/ 100 mol)							
Acetate (Ac)	61.1 ^a^	61.9 ^b^	62.7 ^c^	63.3 ^d^	0.13	<0.001	0.592
Propionate (Pr)	22.9 ^c^	22.5 ^bc^	22.2 ^b^	21.8 ^a^	0.12	<0.001	0.730
Butyrate	12.8 ^b^	12.6 ^b^	12.2 ^a^	12.0 ^a^	0.09	<0.001	0.564
Minor VFA ^4^	3.11 ^b^	2.99 ^ab^	2.89 ^a^	2.91 ^a^	0.041	0.005	0.160
Ac/Pr (mol/mol)	2.69 ^a^	2.78 ^b^	2.85 ^b^	2.93 ^c^	0.022	<0.001	0.952
AFOM (%) ^5^	114 ^a^	119 ^a^	12 5^b^	131 ^b^	1.8	<0.001	0.905
CH_4_ (ml)	6.90	6.69	6.98	7.27	0.201	0.157	0.255
CH_4_/VFA (mL/mmol)	5.40	5.00	4.94	4.90	0.203	0.112	0.375
NH_3_-N (mg/L)	143 ^a^	149 ^b^	157 ^bc^	163 ^c^	3.2	0.001	0.903
Fermentative parameters (24-h incubation)							
pH	6.79	6.79	6.80	6.79	0.010	0.702	0.924
Total VFA (µmol per vial)	2446 ^a^	2489 ^a^	2537 ^b^	2572 ^b^	13.4	<0.001	0.787
Individual VFA (mol/ 100 mol)							
Acetate (Ac)	61.5 ^a^	62.0 ^ab^	62.4 ^b^	62.5 ^b^	0.18	<0.001	0.300
Propionate (Pr)	18.7 ^b^	18.6 ^b^	18.5 ^ab^	18.2 ^a^	0.10	0.004	0.338
Butyrate	15.5 ^b^	15.2 ^a^	15.0 ^a^	15.0 ^a^	0.15	0.026	0.287
Minor VFA ^4^	4.27	4.15	4.13	4.27	0.043	0.996	0.028
Ac/Pr (mol/mol)	3.31 ^a^	3.36 ^ab^	3.41 ^bc^	3.46 ^c^	0.025	<0.001	0.777
AFOM (%) ^5^	220 ^a^	224 ^ab^	228 ^bc^	231 ^c^	1.3	<0.001	0.847
CH_4_ (mL)	14.9	15.0	15.6	14.9	0.31	0.742	0.238
CH_4_/VFA (mL/mmol)	6.10	6.05	6.14	5.79	0.141	0.222	0.306
NH_3_-N (mg/L)	189 ^a^	195 ^b^	199 ^bc^	204 ^c^	2.6	0.002	0.865

^a, b, c^ For each parameter, means not sharing the same superscript differ (*p* < 0.05); ^1^ Mixed diets containing 40% concentrate with 0, 8, 16 or 24% of dried BRO; ^2^ See text for parameters description; DMED: dry matter effective degradability; ^3^ SEM: standard error of the mean. **^4^** Minor VFA included isobutyrate, isovalerate and valerate. ^5^ AFOM: organic matter apparently fermented.

**Table 6:** Degradation parameters of experimental diets with dried broccoli (BRO) used in Experiment 2 ^1^.

Item ^2^	Diet				SEM ^3^	*p* =	
	Control	BRO8	BRO16	BRO24		Lineal	Quadratic
Dry matter							
*a* (%)	33.6 ^a^	43.8 ^b^	40.1 ^b^	44.1 ^b^	0.72	0.003	0.046
*b* (%)	45.6 ^b^	40.4 ^a^	41.4 ^a^	39.0 ^a^	0.63	0.009	0.249
*a* + *b* (%)	79..2	84.2	81.5	83.1	0.61	0.102	0.152
*c* (h^−1^)	0.262 ^b^	0.114 ^a^	0.160 ^a^	0.121^a^	0.0118	0.006	0.037
ED (%)	72.7	73.0	72.8	72.9	0.77	0.939	0.897
Crude protein							
*a* (%)	35.7 ^a^	46.0 ^c^	43.2 ^b^	52.5 ^d^	0.27	< 0.001	0.326
*b* (%)	55.9 ^c^	45.9 ^b^	46.0 ^b^	39.5 ^a^	0.60	< 0.001	0.136
*a* + *b* (%)	91.6	91.8	89.1	95.0	0.45	0.643	0.142
*c* (h^−1^)	0.167	0.138	0.160	0.152	0.0055	0.626	0.295
ED (%)	80.2	81.1	79.6	83.4	0.89	0.093	0.144

^a, b, c^ For each parameter, means not sharing the same superscript differ (*p* < 0.05); ^1^ Mixed diets containing 40% concentrate with 0, 8, 16 or 24% of dried BRO; ^2^ *a:* soluble fraction; *b:* non-soluble potentially degradable fraction; *c*: fractional degradation rate of *b* fraction; ED: effective degradability calculated for a rumen passage rate of 0.042 h^−1^; ^3^ SEM: standard error of the mean.
